# Outcomes After Bilateral Native Nephrectomy in Autosomal Dominant Polycystic Kidney Disease: A Contemporary Single-Centre NHS Cohort Study (2018–2024)

**DOI:** 10.3390/jcm15218456

**Published:** 2026-10-30

**Authors:** Kavyesh Vivek, Nikolaos-Andreas Anastasopoulos, Frank J. M. F. Dor, Vassilios Papalois

**Affiliations:** 1 Imperial College Renal and Transplant Centre, Imperial College Healthcare NHS Trust, Hammersmith Hospital, Du Cane Road, London W12 0HS, UK; kavyesh.vivek2@nhs.net; 2 Department of Surgery and Cancer, Imperial College London, Hammersmith Hospital Campus, Du Cane Road, London W12 0NN, UK; n.anastasopoulos@uoi.gr (N.-A.A.); frank.dor@nhs.net (F.J.M.F.D.)

**Keywords:** autosomal dominant polycystic kidney disease, bilateral native nephrectomy, kidney transplantation, Clavien–Dindo classification, perioperative transfusion, length of stay, National Health Service

## Abstract

**Background/Objectives:** Bilateral native nephrectomy (BNN) is undertaken selectively in autosomal dominant polycystic kidney disease (ADPKD) to relieve symptoms or to create iliac fossa space for transplantation. Contemporary UK National Health Service outcome data are scarce. We describe indications, morbidity and mortality after open BNN at a UK tertiary transplant centre. **Methods:** This is a retrospective descriptive cohort study of 34 consecutive adults with ADPKD undergoing open BNN between August 2018 and September 2024, reported per STROBE, without a comparator group. Pre-specified primary outcomes were length of stay (LoS) and Clavien–Dindo (CD) ≥III complications. Exploratory analyses used regression on log(LoS), with a sensitivity analysis excluding one outlier, and Firth-penalised logistic regression. **Results:** Median age was 55 years (IQR 51–62); 29 patients (85.3%) were dialysis-dependent, and 5 (14.7%) had a functioning allograft. No procedure was concurrent with transplantation. The commonest indication was insufficient space for a kidney graft (21/34, 61.8%). Median LoS was 8 days (IQR 7–10). CD≥III complications occurred in 5/34 (14.7%; 95% CI 6.4–30.1%) and transfusion in 6/34 (17.6%); grade I–II complications were not systematically recorded. There were no in-hospital, 30-day or 90-day deaths. Over a median potential follow-up of 53 months, 15/34 (44.1%) underwent transplantation. Transfusion was associated with longer LoS (β = 0.58; *p* = 0.035), but not after excluding one outlier (*p* = 0.348), and was not significantly associated with CD≥III (OR 4.05; 95% CI 0.54–28.4). No invasive malignancy was found in 29 histopathology reports. **Conclusions:** In this small single-centre series, open BNN was performed mainly to enable transplantation, with no early mortality and a 14.7% CD≥III rate. These descriptive baseline data cannot establish benefit relative to unilateral or no nephrectomy.

## 1. Introduction

Autosomal dominant polycystic kidney disease (ADPKD) is the commonest inherited kidney disorder, with an estimated prevalence of 1 in 400 to 1 in 1000, and a leading cause of kidney failure worldwide. ADPKD accounts for approximately 7–10% of patients listed for kidney transplantation in high-income health systems [1,2,3]. Biallelic loss-of-function in *PKD1* or *PKD2* disrupts the polycystin-1/polycystin-2 complex at the primary cilium, with downstream activation of mTOR and cAMP-mediated cystogenic pathways [4,5]. Diagnosis is most often made by imaging using the unified ultrasonographic criteria of Pei and colleagues, with genetic testing reserved for atypical presentations [6]. Despite a high disease burden, transplantation outcomes in ADPKD are favourable: a 2025 systematic review and meta-analysis of 23 studies reported superior 1-year patient survival (odds ratio [OR] 1.30) and 10-year graft survival (OR 1.60) for ADPKD recipients compared with non-ADPKD recipients, although post-transplant new-onset diabetes is more frequent [7]. Surgical management of the native polycystic kidneys therefore sits within an otherwise efficient transplantation pathway.

Native kidneys in advanced ADPKD are frequently massively enlarged and may cause pain, recurrent cyst infection, haematuria, gastrointestinal compression, and refractory hypertension. Bilateral native nephrectomy (BNN) is performed to relieve symptoms, to resolve diagnostic uncertainty about cystic neoplasia, or to create space for an allograft when iliac fossa volume is inadequate [1,2,3]. Selective rather than routine nephrectomy has been the prevailing principle since Fuller and colleagues established this position in 2005 [8]. Total kidney volume (TKV), particularly the height-adjusted TKV underpinning the Mayo imaging classification, is the most validated determinant of disease severity and operative difficulty [9] and is increasingly used to stratify candidates for surgical and medical intervention. The 2025 ERA Genes & Kidney Working Group consensus statement, summarising 54 studies and 2391 procedures, reports a pooled overall complication rate of 35.1%, a major (Clavien–Dindo [CD]≥III) complication rate of 14.7%, and pooled mortality of 1.5% which rises to 16.7% for emergency operations [1]. The KDIGO 2025 ADPKD guideline endorses these findings, characterises the evidence base as weak, and positions pre-transplant nephrectomy as a last-resort intervention [2].

Three areas remain dispute, the first of which is the indication threshold, particularly graft space considerations versus refractory symptoms [3]. The second is the optimal timing relative to transplantation (pre-transplant, simultaneous, or post-transplant) [10,11,12,13]: the contemporary series of Veroux and colleagues reported that simultaneous unilateral nephrectomy is safe and does not adversely affect graft function [10], and the 2022 systematic review of Xu and colleagues concluded equipoise between staged and concurrent strategies [11]. The third is the surgical approach (open, laparoscopic, hand-assisted, or robotic) [14,15,16,17]. The largest contemporary surgical dataset is the American College of Surgeons National Surgical Quality Improvement Program (ACS-NSQIP) analysis of Bacchus and colleagues (*n* = 823; 479 [58.2%] open and 344 [41.8%] minimally invasive, the latter not subdivided into laparoscopic, hand-assisted, or robotic; 217 [26.4%] bilateral), which reported a 7.0% major complication rate and an apparent advantage for minimally invasive surgery over the open approach (transfusion 7.8% vs. 29.1%; LoS 3 vs. 6 days; OR 0.27 for major complications) [18]. However, the 2026 multivariable analysis of Helal Birjandi and colleagues (*n* = 124) found that this minimally invasive advantage was attenuated after adjustment for kidney size and surgical complexity and that lower pre-operative haemoglobin was independently associated with overall complications (OR 0.74 per g/dL), positioning pre-operative anaemia correction as a directly modifiable risk factor [19]. The 2023 systematic review of Prudhomme and colleagues (3626 patients) further indicates that transcatheter arterial embolisation can achieve 36–49% volume reduction at 3–6 months, providing a non-surgical alternative when the dominant indication is volume rather than parenchymal disease [20].

Bilateral-specific data are sparse. The previously published UK NHS series from Hammersmith Hospital (*n* = 33) and from Guy’s and St Thomas’ (*n* = 31, of whom 28 underwent bilateral nephrectomy) demonstrated stability of graft function and HLA antibody profile after BNN, but the latter reported all-grade morbidity of 65% and mortality of 3% in a historical cohort that pre-dated routine elective scheduling and did not use CD grading [21,22]. A 2024 Irish cohort (*n* = 41; 37% transfusion rate) and the 2025 Hannover–Aachen comparison of pre- and post-transplant timing (*n* = 27) are the most contemporary near-comparable series [23,24]. No randomised trial has been performed.

We therefore evaluated consecutive BNN procedures performed at our UK tertiary transplant centre between 2018 and 2024 with three pre-specified objectives: (i) to characterise indications, peri-operative course, and complications using the CD classification [25]; (ii) to explore factors associated with prolonged LoS and major (CD≥III) morbidity; and (iii) to provide descriptive UK baseline data for comparison with international published cohorts. Reporting follows the Strengthening the Reporting of Observational Studies in Epidemiology (STROBE) statement [26,27].

## 2. Materials and Methods

### 2.1. Study Design

This is a retrospective observational cohort study of consecutive adult patients with ADPKD undergoing BNN at a single UK tertiary transplant centre, conducted as a service evaluation under local clinical governance arrangements. The study is descriptive and involved no comparator group of patients managed with unilateral nephrectomy or without nephrectomy. Reporting follows the STROBE checklist for cohort studies [26,27]; the full mapping is provided in Appendix A.

### 2.2. Setting and Participants

**Figure 1:**
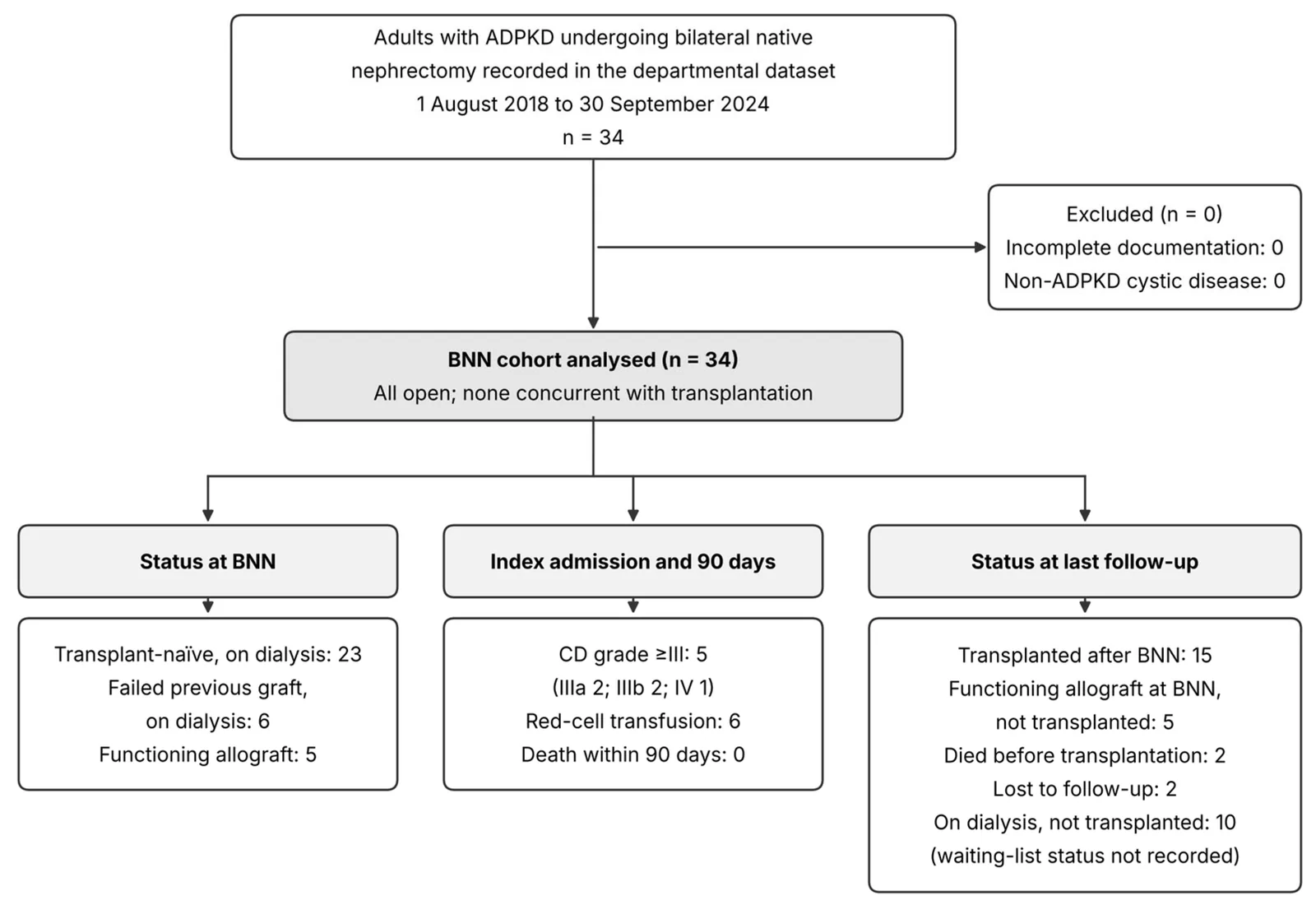
STROBE flow diagram showing identification, exclusion, and outcomes of adults with ADPKD undergoing bilateral native nephrectomy at a UK tertiary transplant centre between August 2018 and September 2024. Categories within status at BNN and within status at last follow-up are mutually exclusive. Unilateral native nephrectomies were not recorded in the departmental dataset and are not shown. ADPKD, autosomal dominant polycystic kidney disease; BNN, bilateral native nephrectomy; CD, Clavien–Dindo.

All native nephrectomy procedures performed in adults with ADPKD between 1 August 2018 and 30 September 2024 at Imperial College Healthcare NHS Trust (Hammersmith Hospital, London) were identified from operative records and the local kidney transplant database and screened. Inclusion criteria were adults (≥18 years) with a clinical diagnosis of ADPKD established by the unified ultrasonographic criteria of Pei and colleagues, or by genetic testing where performed [6], undergoing bilateral (synchronous or sequential same-admission) native nephrectomy for any indication. Exclusion criteria were unilateral nephrectomy alone, non-ADPKD cystic disease, and a missing index admission record. The departmental dataset recorded 34 adults with ADPKD who underwent BNN during the study period. No patient was excluded for incomplete documentation or for non-ADPKD cystic disease, so all 34 were analysed. Unilateral native nephrectomies were not recorded in this dataset. Patient flow is shown in Figure 1.

Selection for BNN was decided by the multidisciplinary transplant team (transplant surgery, nephrology, and radiology), taking into account patient preference; TKV was not used as a formal selection threshold. Insufficient graft space was judged on cross-sectional imaging and clinical examination. The decision to remove both kidneys rather than one was based on multidisciplinary assessment of the size of both kidneys; the case-level rationale was not recorded systematically. Refractory symptoms comprised pain, recurrent cyst infection, haematuria or cyst haemorrhage, and gastrointestinal or respiratory compression despite conservative management. Suspicious or oncologic indications comprised complex cystic lesions on imaging (for example, Bosniak III). All BNN procedures were staged relative to transplantation; none were performed concurrently with graft implantation.

### 2.3. Variables and Data Sources

Demographic, peri-operative, and outcome data were extracted from the institutional electronic health record, operative records, blood-bank records, and the local kidney transplant database. Variables comprised age at BNN; sex; indication category (insufficient graft space; refractory symptoms or complications; suspicious or oncologic; categories were not mutually exclusive); pre-operative dialysis modality; previous kidney transplant and allograft function at the time of BNN; operating surgeon team; LoS in days; peri-operative red cell transfusion (any allogeneic red cell transfusion during the BNN admission); CD grade of the most severe complication, classified per the 2009 five-year validation of the system [25]; native kidney specimen weight and dimensions; histopathological conclusion; subsequent transplantation status and date; graft and patient survival; and date and cause of death where applicable. Follow-up was measured from the date of BNN to death or last documented clinical contact, with data censored on 6 May 2026, the date the dataset was finalised. Because dates of last contact were not recorded, follow-up is reported as potential follow-up to death or data lock, excluding the patient whose date of death was unknown; transplant waiting-list status was not recorded in the dataset. TKV, height-adjusted TKV, pre-operative haemoglobin, intra-operative tranexamic acid administration, and operative time were not consistently recorded; these gaps are addressed in Section 4.5.

Complication grades were adjudicated from contemporaneous operative, radiology, and discharge documentation by two investigators against the 2009 CD definitions [25]. Radiological drainage performed without general anaesthesia was graded IIIa; any return to theatre under general anaesthesia was graded IIIb; complications managed with transfusion or pharmacological therapy alone were graded II. Complications occurring after discharge and within 30 days of BNN were identified from readmission records, outpatient clinic letters, and the electronic health record at our centre; events managed only at referring units would not have been captured. Grade I–II complications were not systematically recorded in the source dataset.

### 2.4. Outcomes

Pre-specified primary outcomes were (i) LoS during the BNN admission and (ii) CD≥III complications within the index admission and 30 days. Secondary outcomes were (i) peri-operative red cell transfusion; (ii) subsequent transplantation and the interval from BNN to transplantation; and (iii) malignant histology on the nephrectomy specimen. Mortality was reported as in-hospital, 30-day, and 90-day mortality, and as all-cause mortality during follow-up with attributed cause. Outcome selection followed contemporary practice and the principles of the Standardised Outcomes in Nephrology–Polycystic Kidney Disease (SONG-PKD) core outcome set [28].

### 2.5. Surgical Approach

All operations were performed by, or under the direct supervision of, a consultant transplant surgeon experienced in ADPKD nephrectomy, using an open transperitoneal midline approach. Adrenal-sparing dissection was the default unless oncologic concern dictated otherwise. Specimens were submitted intact for histopathology. An open approach was used throughout because laparoscopic and robotic BNN had not been adopted at our centre during the study period.

There was no BNN-specific transfusion protocol. Red cell transfusion was at the discretion of the anaesthetic and surgical teams; no fixed haemoglobin threshold was specified for BNN, and pre-transfusion haemoglobin values were not recorded in the dataset. Tranexamic acid and intra-operative cell salvage were not used routinely, and their use was not recorded.

### 2.6. Bias

Selection for BNN reflected multidisciplinary decision-making (transplant surgery, nephrology, and patient preference) and is therefore subject to confounding by indication; sicker or more symptomatic patients are more likely to be referred. Multivariable adjustment was limited by the sample size and the number of events and cannot remove this confounding; analyses of association are therefore exploratory. A sensitivity analysis excluded the most extreme LoS outlier. Information bias was minimised by adjudicating CD grades against explicit criteria from contemporaneous documentation, as described in Section 2.3.

### 2.7. Statistical Analysis

Continuous variables are summarised as median (interquartile range, IQR) given non-Gaussian distributions, with mean and standard deviation reported alongside for age. Categorical variables are summarised as count and percentage with 95% Wilson confidence intervals (CI) where relevant. Two-group comparisons of LoS by transfusion status used the Mann–Whitney U test, with rank-biserial *r* as the effect size. Categorical associations (transfusion versus CD≥III) used Fisher’s exact test. Correlation between age and LoS was assessed by Spearman’s ρ. Exploratory regression analyses comprised (i) ordinary least-squares (OLS) regression on log-transformed LoS, with covariates age, sex, indication group, and transfusion; and (ii) Firth-penalised logistic regression for CD≥III, which reduces small-sample bias with sparse events [29,30], fitted for transfusion alone and with age (per 10 years) as the only covariate given five events, with 95% CIs from the profile penalised likelihood. Follow-up is summarised as median (IQR; range). A post hoc sensitivity analysis repeated the principal analyses after excluding the patient whose histology described acquired cystic kidney disease.

A pre-specified sensitivity analysis repeated all LoS analyses after excluding a single extreme outlier (LoS 125 days) attributable to a multi-organ adverse course; both primary and sensitivity estimates are reported. Two-sided *p* values < 0.05 were considered statistically significant. Given the small sample and the small number of CD≥III events (*n* = 5), all analyses of association are exploratory and hypothesis-generating, and none is adjusted for kidney size. Missing data were not imputed; analyses are complete-case for each variable, with denominators stated. Analyses were performed in Python 3.11 using SciPy 1.17 and statsmodels 0.14; Firth regression was implemented in Python by penalised maximum likelihood.

### 2.8. Ethical Approval

This study was conducted as a retrospective service evaluation/audit. Formal research ethics committee approval was therefore not required under UK Health Research Authority guidance. All data were managed in accordance with local information governance and the UK Data Protection Act 2018.

## 3. Results

### 3.1. Cohort Description

A total of 34consecutive patients underwent BNN over the 74-month study period (Figure 1). Median age was 55 years (IQR 52–62; mean 55.6 ± 9.4; range 28–74), and 19/34 (55.9%) were male. Some 29 patients (85.3%) were dialysis-dependent at the time of BNN, and 11 (32.4%) had a previous kidney transplant. At BNN, 23 patients (67.6%) were transplant-naïve and dialysis-dependent, 6 (17.6%) had a failed previous transplant and had returned to dialysis, and 5 (14.7%) had a functioning allograft. Indications were insufficient graft space in 21/34 (61.8%), refractory symptoms or complications in 12/34 (35.3%), and suspicious or oncologic in 2/34 (5.9%); 1 patient had both insufficient graft space and refractory symptoms and is counted in both categories. Of the 21 patients with insufficient graft space, 17 were transplant-naïve, 3 had a failed previous transplant, and 1 had a functioning allograft (kidneys measuring 260 mm and 335 mm). Ex vivo specimen dimensions were recorded for both kidneys in 19 of these 21 patients: the median maximum specimen dimension was 235 mm (IQR 205–263; range 152–405), and the smaller kidney measured at least 200 mm in 14 of 19 and at least 180 mm in 16 of 19. Of the remaining four patients with a functioning allograft, three underwent BNN for refractory symptoms and one for a suspicious lesion. Median potential follow-up from BNN was 53 months (IQR 38–76; range 19–92). Baseline characteristics are summarised in Table 1.

### 3.2. Operative Outcomes

Median LoS was 8 days (IQR 7–10; range 5–125). Excluding a single extreme outlier (LoS 125 days, attributable to the multi-organ adverse course described below), median LoS was unchanged at 8 days (IQR 7–10; range 5–20). Peri-operative red cell transfusion was administered to 6/34 patients (17.6%; 95% CI 8.3–33.5%).

### 3.3. Complications

CD≥III complications occurred in 5/34 patients (14.7%; 95% CI 6.4–30.1%). Two patients (5.9%) underwent radiological drainage without general anaesthesia (CD IIIa), one for ascites and one for a lymphocele.

Two patients (5.9%) required a return to theatre under general anaesthesia (CD IIIb). The first underwent excision of an inflammatory granuloma at the drain exit site on the abdominal wall, which developed after removal of the operative drain and did not resolve with conservative management; the same patient had also had post-operative intra-abdominal collections and received a red cell transfusion during the index admission. The second underwent relaparotomy with operative drainage of an intra-abdominal collection. Neither return to theatre was for haemorrhage, vascular injury, or visceral perforation, and neither patient required critical care escalation.

One patient (2.9%) sustained a life-threatening multi-organ adverse course (CD IV) comprising haemorrhagic stroke and sepsis requiring multiple radiologically guided drainages; this patient accounted for the LoS of 125 days and survived to discharge. There were no CD V events (Table 2).

One further patient sustained intra-operative haemorrhage managed with red cell transfusion alone, without radiological or operative intervention; this was graded CD II and is therefore not included in the CD≥III denominator, although it was recorded as a major peri-operative event in the source dataset. A post hoc definition including this event would yield a CD≥III rate of 6/34 (17.6%).

### 3.4. Univariable Associations

Transfused patients had a longer LoS than non-transfused patients (medians 10 vs. 7.5 days; Mann–Whitney U = 131.0; *p* = 0.032; rank-biserial *r* = 0.56). Excluding the 125-day outlier attenuated this association to a non-significant trend (medians 10 vs. 7.5; U = 103.0; *p* = 0.096; *r* = 0.47), reflecting the influence of a single high-leverage observation in a small sample. Age correlated weakly and non-significantly with LoS overall (Spearman ρ = 0.26; *p* = 0.136; *n* = 34) and the correlation remained weak and not statistically significant after excluding the outlier (ρ = 0.33; *p* = 0.059; *n* = 33). CD≥III complications were more frequent among transfused patients (2/6 vs. 3/28), but the association did not reach statistical significance (Fisher’s exact OR 4.17; *p* = 0.205).

### 3.5. Exploratory Regression Analyses

In OLS regression of log(LoS) on age, sex, indication group, and transfusion (*n* = 34; df_resid_ = 28; R^2^ = 0.18), peri-operative transfusion was associated with longer hospitalisation (β = 0.579; SE 0.262; t = 2.21; *p* = 0.035), corresponding to an approximate 78% relative increase in median LoS. No other covariate reached statistical significance. In a sensitivity model excluding the LoS outlier (*n* = 33; R^2^ = 0.17), the transfusion coefficient was attenuated and not statistically significant (β = 0.155; *p* = 0.348), indicating that the association in the primary model depended on a single observation.

Given five CD≥III events, the association between transfusion and CD≥III was examined with Firth-penalised logistic regression. Transfusion was not significantly associated with CD≥III, either unadjusted (OR 4.05; 95% CI 0.54–28.35; *p* = 0.162) or adjusted for age (OR 3.65; 95% CI 0.50–24.71; *p* = 0.190). Age was not associated with CD≥III (OR 0.73 per 10 years; 95% CI 0.27–2.11; *p* = 0.538). These intervals are compatible with both no association and a large association. Estimates are summarised in Table 3. In the sensitivity analysis excluding the patient whose histology described acquired cystic kidney disease (*n* = 33), estimates were materially unchanged: CD≥III 5/33 (15.2%), transfusion–LoS β = 0.579 (*p* = 0.039), and Firth OR for transfusion and CD≥III 3.89 (95% CI 0.52–27.28).

### 3.6. Follow-Up and Transplantation Status After BNN

Median potential follow-up from BNN was 53 months (IQR 38–76; range 19–92). Some 15 of 34 patients (44.1%; 95% CI 28.9–60.5%) underwent transplantation during follow-up (14 first and 1 repeat transplant) at a median of 365 days (IQR 238–642; range 102–1364). All 15 were dialysis-dependent at BNN (15/29, 51.7%). No graft loss was recorded among the 14 recipients with documented graft status; graft status had not yet been recorded for the most recent recipient, transplanted in October 2025. Three recipients died with a functioning graft (Section 3.7). Of the 19 patients not transplanted after BNN, 5 had a functioning allograft at BNN, 2 died before transplantation, 2 were lost to follow-up after transfer of care, and 10 remained dialysis-dependent without transplantation at last recorded follow-up (Table 4). Waiting-list status (active, suspended, or under evaluation) was not recorded for these 10 patients and could not be retrieved retrospectively; whether the 2 patients who died before transplantation were listed at the time of death is likewise unknown. Of the 10, 4 had a failed previous transplant, follow-up status was uncertain in 1, and the median interval from BNN to data lock was 51 months (range 19–91); 6 had exceeded the longest interval from BNN to transplantation observed among recipients (1364 days).

### 3.7. Mortality and Causes of Death

There were no deaths during the index admission, within 30 days, or within 90 days of BNN (in-hospital, 30-day and 90-day mortality all 0%). Six patients (17.6%) died during subsequent follow-up. Among the five patients with a documented date of death, the earliest death occurred 575 days (19 months) after BNN, and the median interval from BNN to death was 859 days (range 575–1270). The sixth death was reported to have occurred in 2022, between 3 and 15 months after BNN; the 90-day mortality estimate for this patient therefore rests on the reported year of death. No documented death was attributable to the nephrectomy, to a nephrectomy-related complication, or to the index admission.

Cause of death was documented in five patients: metastatic pancreatic carcinoma, ischaemic heart disease, COVID-19, endometrial carcinoma, and heart failure with hospital-acquired pneumonia. The sixth patient was lost to follow-up after transfer of care and was subsequently reported to have died in 2022; neither the date nor the cause of death could be verified from institutional records, and this death is therefore reported as of undetermined cause. Three of the six patients who died had been transplanted after BNN and died with a functioning graft; the remaining three had not been transplanted after BNN. Individual data are shown in Table 5.

### 3.8. Histopathology

Histopathology reports were available for 29 of 34 patients. Twenty-seven reports described features consistent with ADPKD; one described acquired cystic kidney disease (this patient was included on the basis of the recorded clinical diagnosis of ADPKD; excluding the patient did not materially change any estimate, Section 3.5), and one described end-stage kidney disease without further characterisation. No invasive renal malignancy was identified in any of the 29 reports. Papillary or tubulopapillary adenomata were reported in eight specimens, and one specimen showed multifocal micropapillary epithelial hyperplasia of cyst linings without invasive malignancy. Specimen weights were recorded for 35 kidneys from 18 patients and ranged from 629 g to 4894 g, excluding one 36 g kidney from the patient with acquired cystic kidney disease (Table 6).

## 4. Discussion

### 4.1. Principal Findings

In this single-centre UK cohort of 34 consecutive patients with ADPKD undergoing open BNN, the leading indication was insufficient graft space (61.8%), median LoS was 8 days, and 14.7% of patients had a CD≥III complication. There were no deaths during the index admission or within 90 days, and no documented death during follow-up was related to the procedure. Returns to theatre were infrequent (2/34, 5.9%) and neither of the two was for haemorrhage. Peri-operative red cell transfusion (17.6%) was associated with longer LoS in the primary model, but this association depended on a single outlier and should be regarded as exploratory; its association with CD≥III was imprecise and not statistically significant. Without a comparator group, these data describe outcomes after BNN and cannot establish its benefits or risks relative to unilateral or no nephrectomy. They provide contemporary UK baseline data alongside the 2025 ERA consensus and the largest population-based dataset to date [1,18].

### 4.2. Comparison with the Existing Literature

Our results sit within the contemporary international range (Appendix B, Table A2), although cross-series comparison is limited by differences in inclusion criteria, approach, era, and grading. The CD≥III rate of 14.7% is identical to the ERA pooled estimate of 14.7% [1] and comparable to the 27-patient bilateral series of Tessmer and colleagues, in which CD IV occurred in 5% of post-transplant BNN [24]. It is higher than the 7.0% major complication rate reported by Bacchus and colleagues in the largest contemporary dataset (*n* = 823; ACS-NSQIP) [18], although that estimate includes 41.8% minimally invasive and 73.6% unilateral procedures and is not directly comparable with a series in which all operations were open and bilateral. The registry also does not record operative technique or kidney volume. Our median LoS of 8 days is shorter than the pre-transplant medians of 10–19 days in continental European series [14,24], similar to the 9-day median of the earlier Hammersmith series and the contemporary Irish cohort [21,23].

Both returns to theatre arose from drain-site and collection-related problems rather than from bleeding or technical failure of the nephrectomy. Drain-site granuloma, a foreign-body inflammatory response to prolonged drain contact, is rarely enumerated in nephrectomy series, yet it accounted for half the reoperative burden in this cohort. Series that report only bleeding, visceral injury, and death will therefore understate the CD IIIb rate relative to series that adjudicate every return to theatre. Whether shorter drain dwell time reduces this complication is not assessed here.

The transfusion rate (17.6%) is lower than the 22.8% reported with simultaneous ipsilateral nephrectomy in the matched-pairs analysis of Jänigen and colleagues [13], substantially lower than the 85.7% rate in the pre-transplant subgroup of Tessmer and colleagues [24] and approximately half the 37% rate of the Irish cohort of Hogan and colleagues [23]. It remains more than twice the 7.8% benchmark for minimally invasive ADPKD nephrectomy in ACS-NSQIP and approximately half the 29.1% rate reported in that registry for open procedures [18]. The improvement compared with the 42% transfusion rate of the previous Hammersmith era and the historical morbidity and mortality of the Guy’s and St Thomas’ series (65% all-grade morbidity, 3% mortality, *n* = 31) [21,22] may reflect changes in patient selection and elective scheduling, although differences in era, case mix, and complication grading limit this comparison. Deaths with a documented date occurred no earlier than 19 months after BNN (the sixth occurred 3 to 15 months after BNN) and, where the cause was documented, were attributable to malignancy, cardiac disease, and infection in a population with substantial competing risk; no documented death was procedure-related.

Regarding indication and timing, our 61.8% graft space indication aligns with the ERA pooled distribution (42.6%) and with the earlier Hammersmith series (59%) [1,21]. Current recommendations favour a selective and, where feasible, unilateral approach [1,2]. In our cohort, the choice of BNN rather than unilateral nephrectomy for graft space reflected multidisciplinary assessment of the size of both kidneys rather than a protocol-defined threshold. In this group, the smaller kidney measured at least 200 mm in 14 of 19 patients with recorded specimen dimensions, consistent with bilateral encroachment on the iliac fossae, although ex vivo specimen length is an imperfect surrogate for in situ volume. Because no unilateral comparator was available, we cannot determine whether unilateral nephrectomy would have sufficed in some of these patients. All procedures were staged, so our data do not inform the simultaneous strategy. Simultaneous unilateral [10] and bilateral [31] nephrectomy with transplantation appear safe, and staged and concurrent strategies are in equipoise, with economic and LoS advantages for simultaneous approaches where logistics permit [11,12]. Some 15 of 34 patients (44.1%; 15/29 of those without a functioning allograft) proceeded to transplantation at a median of 365 days, comparable with post-nephrectomy listing intervals reported by Casteleijn and colleagues [32]. Long-term graft outcomes after pre-transplant BNN appear preserved: in the 112-patient cohort of Rosenberg and colleagues, pre-transplant nephrectomy was not associated with increased mortality or long-term complications [33], consistent with the absence of documented procedure-related deaths in our cohort and with the favourable post-transplant outcomes reported for ADPKD recipients [7].

The absence of invasive renal malignancy contrasts with the pooled per-person renal cell carcinoma (RCC) prevalence of 5.7% in the meta-analysis of Drake and colleagues [34], the 12.5% incidence reported by Mansbridge and colleagues [35], and the 7% incidence in the Irish cohort [23]. Our zero RCC rate is closer to the 1.3% incidence in the Guy’s and St Thomas’ series [22] and may reflect differences in immunosuppression exposure and case mix between series; with 29 histopathology reports, the 95% CI for a zero rate extends to 11.7%. Papillary or tubulopapillary adenomata (recognised as indolent or pre-malignant findings) in eight specimens support continued routine histopathological examination.

### 4.3. Implications and Areas for Prospective Evaluation

Three implications follow, each limited by the descriptive design. First, transfusion was common, but its association with LoS depended on a single outlier, and its association with major morbidity was imprecise. Transfusion is plausibly a marker of operative difficulty (larger kidneys, denser adhesions, longer operating time) as much as an independent hazard, and residual confounding by kidney size, which we could not measure, would bias the observed associations away from the null. Lower pre-operative haemoglobin has been independently associated with complications after native nephrectomy (OR 0.74 per g/dL) [19], and frameworks for peri-operative anaemia management [36] and patient blood management [37] are established in other surgical settings. None of these measures were evaluated in this cohort, and their effect on transfusion and morbidity after BNN requires prospective evaluation.

Prophylactic tranexamic acid and intra-operative cell salvage are candidates for such evaluation. In meta-analyses of randomised trials in elective abdominal and major oncological pelvic surgery, tranexamic acid reduced blood loss and red cell transfusion (OR 0.40–0.43) without an increase in venous thromboembolism [38,39], and wider surgical use has been advocated [40]. Cell salvage reduced allogeneic transfusion in a Cochrane review of predominantly cardiac and orthopaedic trials at high risk of bias (risk ratio 0.62; 95% CI 0.55–0.70) [41], and NICE guidance supports both measures where substantial blood loss is anticipated [42]. Neither was used routinely or recorded in this cohort, ADPKD-specific data are absent, and infected cyst fluid and suspected malignancy are relative contraindications to cell salvage in a subset of patients.

The transplant relevance of transfusion is heightened by its link with HLA sensitisation: in the review of Willicombe and Roberts, de novo HLA antibodies developed in 62% of transfused waitlist patients and 23% of previously non-sensitised patients [43]. In our cohort, paired pre- and post-BNN calculated reaction frequency (cRF) was available for only three of the six transfused patients and was unchanged at 0% in each, so this effect could not be quantified.

Second, there were no early deaths after elective BNN in this cohort, whereas pooled mortality after emergency BNN reached 16.7% [15]; this indirect comparison is consistent with, but does not demonstrate, a benefit of elective scheduling. Complete reporting of returns to theatre, rather than reporting restricted to bleeding and visceral injury, would improve comparability between series.

Third, the absence of invasive malignancy, set against the recognised pooled RCC prevalence of approximately 5% [34], supports routine histopathological examination but does not, on these data, justify pre-emptive BNN solely for malignancy surveillance. Where the dominant indication is volume rather than parenchymal disease or symptoms, transcatheter arterial embolisation deserves consideration, with reported volume reductions of 36–49% at 3–6 months [20]. Patient-reported outcomes are also relevant: in the largest dedicated study to date (*n* = 276), pre-transplant nephrectomy significantly improved post-transplant ADPKD Impact Scale (ADPKD-IS) and 36-Item Short Form Survey (SF-36) physical scores, with bilateral procedures associated with greater physical quality-of-life benefit than unilateral nephrectomy [44]. Our data describe outcomes after open BNN but cannot show that open BNN should be preferred to minimally invasive or robotic approaches [16,17], which were not used at our centre during the study period.

### 4.4. Strengths

Strengths include consecutive sampling across a defined period at a high-volume UK tertiary transplant centre; explicit, criterion-based re-adjudication of CD grades rather than acceptance of contemporaneous coding [25]; complete ascertainment of in-hospital, 30-day, and 90-day mortality, with cause and interval for later deaths where documented; and a pre-specified sensitivity analysis. This is, to our knowledge, the only contemporary CD-graded NHS series of bilateral native nephrectomy.

### 4.5. Limitations

Several limitations should temper interpretation. First, there was no comparator group of patients managed with unilateral nephrectomy or without nephrectomy, so the study cannot estimate the relative benefits and risks of BNN; it describes outcomes in patients selected for BNN. Because unilateral native nephrectomies were not recorded in the departmental dataset, we also cannot report the proportion of native nephrectomies that were bilateral. Second, the sample size (*n* = 34) and the small number of CD≥III events (*n* = 5) constrain statistical power and produce wide confidence intervals; the CD≥III analysis is underpowered, and its non-significant result should not be read as evidence of no association. Third, this is a retrospective single-centre cohort, subject to confounding by indication (sicker or more symptomatic patients are preferentially selected for BNN, which biases complication estimates upwards relative to an unselected population) and to information bias from documentation variability. Grade I–II complications were not systematically captured and were noted only incidentally (for example, infected collections and wound infection), so overall morbidity is underestimated. In the ERA pooled analysis, the overall complication rate (35.1%) was more than twice the CD≥III rate (14.7%) [1], indicating that most complications after nephrectomy are grade I–II; our data therefore cannot be compared with series reporting all-grade morbidity, and unrecorded minor complications (for example, wound infection or ileus) may account for part of the variation in LoS. Complications managed after discharge at referring units may also have been missed, which would bias the CD≥III rate downwards.

Fourth, several variables of recognised prognostic value were not consistently recorded and could not be modelled. TKV and height-adjusted TKV, the validated predictors of disease severity and the variables most directly relevant to surgical planning [9], are absent, and ex vivo specimen dimensions are only a partial surrogate; this is the single most important data gap, and their omission means that residual confounding by kidney size almost certainly persists in the transfusion–outcome models, in the direction of overstating the transfusion effect. Pre-operative haemoglobin, recently identified as an independent predictor of complications [19], was not systematically captured. Operative time, estimated blood loss, intra-operative haemoglobin nadir, and critical care utilisation were similarly incomplete. Tranexamic acid was not routinely administered during the study period and neither its use nor that of intra-operative cell salvage was recorded.

Fifth, the transfusion–LoS effect was sensitive to a single high-leverage outlier (LoS 125 days), and the corresponding estimate is therefore unstable. Sixth, all operations were performed open, so our complication and transfusion estimates should not be taken as representative of contemporary minimally invasive practice [18,19]. Seventh, paired pre- and post-BNN cRF was recorded in only 15 patients (3 of them transfused) and was unchanged in all 15, which precludes direct quantification of any allosensitisation effect on subsequent graft access [43]. Eighth, dialysis modality continuity, in particular whether peritoneal dialysis patients could continue peritoneal dialysis after BNN, was not captured. Ninth, one death could not be verified for date or cause, and follow-up for two further patients was incomplete after transfer of care; ascertainment of later deaths and transplant outcomes is therefore incomplete, waiting-list status of patients not transplanted was not recorded, and the reported causes of death rely on clinical documentation rather than death certification data. Finally, the small sample, single-centre setting, and exclusive use of an open approach limit generalisability to other centres and to minimally invasive practice; thresholds for nephrectomy and indication framing also differ across health systems [1,2].

### 4.6. Future Directions

These data motivate four lines of further work, the first of which is a prospective evaluation of a structured peri-operative blood management pathway for BNN (pre-operative anaemia correction, prophylactic tranexamic acid, and cell salvage where appropriate) with LoS, complications, and allosensitisation as outcomes; given the trial evidence in other major abdominal and pelvic surgery [38,39,40], a pragmatic randomised trial of tranexamic acid in BNN would test this directly. The second is an NHS blood and transplant-linked analysis of graft and patient survival after BNN, including comparison with unilateral nephrectomy and with pre-transplant, simultaneous, and post-transplant timing strategies. The third is a prospective audit of laparoscopic and robotic BNN where kidney size and surgeon expertise permit, with adjustment for case complexity and TKV [1,16,17,19]. The fourth is incorporation of patient-reported outcome measures, in particular ADPKD-specific quality-of-life domains [44], within the SONG-PKD core outcome set framework [28].

## 5. Conclusions

In this small single-centre UK cohort of 34 consecutive patients with ADPKD, open BNN was performed predominantly to enable transplantation and was followed by no in-hospital, 30-day, or 90-day mortality; a 14.7% CD≥III complication rate; and a 17.6% transfusion rate. No documented death during follow-up was related to the procedure, although the date and cause of one death could not be verified. Transfusion was associated with longer LoS, but this association depended on a single outlier, and its association with major morbidity was imprecise. Grade I–II complications were not recorded, so overall morbidity is underestimated. Without a comparator group, these data cannot establish the benefit of BNN over unilateral or no nephrectomy, and their generalisability is limited by the sample size, the single-centre setting, and the exclusive use of an open approach. They provide baseline data against which prospective evaluation of blood management strategies and minimally invasive approaches can be measured.

## Figures and Tables

**Table 1:** Baseline, operative, and follow-up characteristics (*n* = 34).

Variable	Value
Age (years), median (IQR)	55 (51–62)
Age, mean ± SD; range	55.6 ± 9.5; 28–74
Male sex, *n* (%)	19 (55.9)
Dialysis-dependent at BNN, *n* (%)	29 (85.3)
Previous kidney transplant, *n* (%); functioning at BNN, *n* (%)	11 (32.4); 5 (14.7)
Status at BNN: transplant-naïve, dialysis-dependent, *n* (%)	23 (67.6)
Status at BNN: failed previous transplant, dialysis-dependent, *n* (%)	6 (17.6)
Status at BNN: functioning allograft, *n* (%)	5 (14.7)
Indication: insufficient graft space, *n* (%)	21 (61.8)
Indication: refractory symptoms/complications, *n* (%)	12 (35.3)
Indication: suspicious/oncologic, *n* (%)	2 (5.9)
Length of stay (days), median (IQR; range)	8 (7–10; 5–125)
Length of stay excluding outlier (*n* = 33), median (IQR; range)	8 (7–10; 5–20)
Peri-operative red cell transfusion, *n* (% [95% CI])	6 (17.6 [8.3–33.5])
Clavien–Dindo≥III, *n* (% [95% CI])	5 (14.7 [6.4–30.1])
Return to theatre (CD IIIb), *n* (%)	2 (5.9)
In-hospital, 30-day and 90-day mortality, *n* (%)	0 (0)
Transplantation after BNN, *n* (% [95% CI])	15 (44.1 [28.9–60.5])
Time from BNN to transplantation (days), median (IQR; range)	365 (238–642; 102–1364)
Invasive renal malignancy on histology, *n*/*N*	0/29
Potential follow-up from BNN (months), median (IQR; range)	53 (38–76; 19–92)

Indication categories are not mutually exclusive; one patient is counted under both insufficient graft space and refractory symptoms. Status at BNN categories are mutually exclusive. Wilson 95% confidence intervals are reported for principal binary outcomes. BNN, bilateral native nephrectomy; CD, Clavien–Dindo; CI, confidence interval; IQR, interquartile range; SD, standard deviation.

**Table 2:** Complications by Clavien–Dindo grade (*n* = 34).

Grade	*n* (%)	Events
I–II (no radiological or operative intervention)	1 (2.9)	Intra-operative haemorrhage managed with red cell transfusion alone
IIIa (radiological intervention without general anaesthesia)	2 (5.9)	Ascites requiring IR drain (×1); lymphocele requiring IR drain (×1)
IIIb (return to theatre under general anaesthesia)	2 (5.9)	Excision of drain-site granuloma (×1); relaparotomy with drainage of intra-abdominal collection (×1)
IV (life-threatening; single- or multi-organ dysfunction)	1 (2.9)	Haemorrhagic stroke and sepsis with multiple IR drainages (LoS 125 days)
V (death)	0 (0)	—
**Total CD≥III**	**5 (14.7)**	—

Grade I–II events other than the intra-operative haemorrhage shown were not systematically captured; this row is therefore not a complete count of grade I–II complications, and overall morbidity is underestimated. Complications were recorded for the index admission and within 30 days of BNN. CD, Clavien–Dindo; IR, interventional radiology; LoS, length of stay.

**Table 3:** Exploratory univariable and regression analyses for primary outcomes.

Analysis	Estimate (95% CI)	*p*
Mann–Whitney U: LoS by transfusion (all, *n* = 34)	Medians 10 vs. 7.5; U = 131.0; *r* = 0.56	0.032
Mann–Whitney U: LoS by transfusion (excluding LoS = 125; *n* = 33)	Medians 10 vs. 7.5; U = 103.0; *r* = 0.47	0.096
Spearman ρ: age vs. LoS (all, *n* = 34)	ρ = 0.26	0.136
Spearman ρ: age vs. LoS (*n* = 33)	ρ = 0.33	0.059
Fisher’s exact: transfusion vs. CD≥III	OR 4.17	0.205
OLS log(LoS) ~ age + sex + indication + transfusion (*n* = 34; R^2^ = 0.18): transfusion β	β = 0.579 (0.043 to 1.114)	0.035
OLS log(LoS) sensitivity (*n* = 33): transfusion β	β = 0.155 (−0.177 to 0.487)	0.348
Firth logistic CD≥III ~ transfusion (*n* = 34; 5 events): transfusion OR	OR 4.05 (0.54 to 28.35)	0.162
Firth logistic CD≥III ~ transfusion + age per 10 years (*n* = 34; 5 events): transfusion OR	OR 3.65 (0.50 to 24.71)	0.190

All analyses are exploratory. Firth-penalised logistic regression with 95% CIs from the profile penalised likelihood; intervals are wide because of sparse data (five CD≥III events). The OLS transfusion coefficient was not statistically significant after exclusion of the LoS outlier. β, regression coefficient on the log scale; CD, Clavien–Dindo; CI, confidence interval; LoS, length of stay; OLS, ordinary least squares; OR, odds ratio; *r*, rank-biserial correlation; ρ, Spearman’s rank correlation coefficient.

**Table 4:** Status at last follow-up after BNN (*n* = 34).

Status	*n* (%)	Details
Transplanted after BNN	15 (44.1)	14 first and one repeat transplant; 3 later died with a functioning graft
Functioning allograft at BNN; transplantation not required	5 (14.7)	One died (COVID-19, 859 days after BNN)
Died before transplantation	2 (5.9)	Ischaemic heart disease (641 days); undetermined cause (death reported 2022)
Lost to follow-up after transfer of care	2 (5.9)	Including one patient whose care transferred overseas
Dialysis-dependent, not transplanted at last follow-up	10 (29.4)	Waiting-list status not recorded; four with a failed previous transplant; median 51 months (range 19–91) from BNN to data lock
**Total**	**34 (100)**	–

Categories are mutually exclusive. BNN, bilateral native nephrectomy.

**Table 5:** Deaths during follow-up (*n* = 6 of 34).

Case	Age at BNN (Years)	Transplanted After BNN	Interval BNN to Death (Days)	Cause of Death
A	62	Yes	575	Metastatic pancreatic carcinoma
B	55	No	641	Ischaemic heart disease
C	65	No	859	COVID-19
D	70	Yes	971	Endometrial carcinoma
E	65	Yes	1270	Heart failure with hospital-acquired pneumonia
F	63	No	Not documented ^1^	Undetermined ^1^

^1^ Patient lost to follow-up after transfer of care; death reported to have occurred in 2022 but neither date nor cause verifiable from institutional records. No death occurred during the index admission or within 90 days of BNN. BNN, bilateral native nephrectomy.

**Table 6:** Histopathological summary (*n* = 29 patients with histopathology reports).

Finding	*n*/29
Features consistent with ADPKD	27
Acquired cystic kidney disease	1
End-stage kidney disease, not otherwise specified	1
Invasive renal cell carcinoma	0
Papillary or tubulopapillary adenoma(ta)	8
Multifocal micropapillary epithelial hyperplasia (no invasive malignancy)	1
Specimen weight, range (g per kidney)	629–4894 (35 kidneys, 18 patients) ^1^

Histopathology reports were unavailable for five patients. ^1^ Excludes one 36 g kidney with features of acquired cystic kidney disease. ADPKD, autosomal dominant polycystic kidney disease.

## Data Availability

The data presented in this study are available on request from the corresponding author. The data are not publicly available because they derive from an NHS single-centre clinical dataset in which the small cohort size creates a risk of patient re-identification.

## Abbreviations

The following abbreviations are used in this manuscript:

ACS-NSQIP	American College of Surgeons National Surgical Quality Improvement Program
ADPKD	Autosomal dominant polycystic kidney disease
ADPKD-IS	ADPKD Impact Scale
BNN	Bilateral native nephrectomy
CD	Clavien–Dindo
CI	Confidence interval
cRF	Calculated reaction frequency
ERA	European Renal Association
Hb	Haemoglobin
HLA	Human leukocyte antigen
IQR	Interquartile range
IR	Interventional radiology
KDIGO	Kidney Disease: Improving Global Outcomes
LoS	Length of stay
NHS	National Health Service
OLS	Ordinary least squares
OR	Odds ratio
RCC	Renal cell carcinoma
SF-36	36-Item Short Form Survey
SONG-PKD	Standardised Outcomes in Nephrology–Polycystic Kidney Disease
STROBE	Strengthening the Reporting of Observational Studies in Epidemiology
TKV	Total kidney volume

## Appendix A

**Table A1:** STROBE checklist mapping (cohort version). Reporting follows the STROBE statement [26].

Item	STROBE Recommendation	Manuscript Location
1a	Indicate study design in title or abstract	Title; Abstract (“retrospective cohort study”)
1b	Provide informative abstract	Abstract: Background/Objectives, Methods, Results, Conclusions
2	Background/rationale	Section 1, paragraphs 1–4
3	Objectives/hypotheses	Section 1, paragraph 5 (three pre-specified objectives)
4	Study design	Section 2.1
5	Setting, locations, dates	Section 2.2
6a	Eligibility criteria, sources, methods of selection	Section 2.2
6b	Matched studies: matching criteria	Not applicable (unmatched cohort)
7	Outcomes, exposures, predictors, confounders, effect modifiers	Section 2.3 and Section 2.4
8	Data sources/measurement	Section 2.3
9	Bias	Section 2.6; Section 4.5
10	Sample size	Section 2.7 (no a priori power calculation; consecutive sample)
11	Quantitative variables	Section 2.7
12a	Statistical methods	Section 2.7
12b	Subgroups/interactions	Section 2.7; Section 3.4 and Section 3.5
12c	Missing data	Section 2.7 (complete case); Section 4.5
12d	Loss to follow-up	Section 3.6; Table 4; Section 4.5
12e	Sensitivity analyses	Section 2.7; Section 3.4 and Section 3.5
13a	Numbers at each stage	Section 2.2; Figure 1
13b	Reasons for non-participation	Section 2.2; Figure 1
13c	Flow diagram	Figure 1
14a	Characteristics of participants	Table 1; Section 3.1
14b	Missing data per variable	Reported in line
14c	Follow-up time	Section 3.1; Table 1; Section 3.6
15	Outcome data	Table 1, Table 2, Table 4, Table 5 and Table 6; Section 3.2, Section 3.3, Section 3.6 and Section 3.7
16a	Unadjusted estimates	Section 3.4; Table 3
16b	Adjusted estimates	Section 3.5; Table 3 (exploratory)
16c	Category boundaries when continuous variables categorised	Indication groups defined in Section 2.3; LoS analysed continuously
17	Other analyses	Sensitivity analysis excluding LoS = 125 outlier; post hoc CD≥III definition (Section 3.3)
18	Key results summarised	Section 4.1
19	Limitations	Section 4.5
20	Interpretation	Section 4.2 and Section 4.3
21	Generalisability	Section 4.5
22	Funding	Funding statement

## Appendix B

**Table A2:** Comparison of the present cohort with key published bilateral native nephrectomy cohorts.

Study	Country/Setting	n (BNN)	Approach	LoS (Days)	Transfusion (%)	CD≥III (%)	Mortality (%)
**Present series**	UK NHS (Hammersmith)	34	Open	8 (median)	17.6	14.7	0 (in-hospital, 30- and 90-day)
ERA consensus 2025 [1]	International, 54 studies	2391 procedures	Mixed	Variable	Variable	14.7	1.5; up to 16.7 (emergency)
Bacchus 2026 [18]	USA (ACS-NSQIP)	823 (217 bilateral)	Mixed (MIS vs. open)	Open 6/MIS 3	Open 29.1/MIS 7.8	7.0	0.5
Helal Birjandi 2026 [19]	Multicentre	124 episodes	Mixed; multivariable	NR	NR	Pre-op Hb OR 0.74 per g/dL	NR
Tessmer 2025 [24]	Germany (Hannover/Aachen)	27 (7 pre-, 20 post-Tx)	Open	Pre 19/Post 12	Pre 85.7/Post 0	Pre 43/Post 5	0
Maxeiner 2019 [14]	Germany (Charité)	121 (unilateral only)	Open retroperitoneal	Pre 7/Post 6	NR	NR	Pre 3.4/Post 0
Lubennikov 2021 [15]	Russia	108	Open vs. laparoscopic	Open 12.5/Lap 8	Similar	NR	16.7 (emergent)
Bellini 2019 [21]	UK NHS (Hammersmith, earlier era)	33	Open	9 (median)	42	Not graded	1/33 (3; non-perioperative)
Patel 2011 [22]	UK NHS (Guy’s and St Thomas’)	31 (28 bilateral)	Open/laparoscopic	Open longer (*p* = 0.003)	NR	Not graded; 65 (all-grade)	3
Hogan 2024 [23]	Ireland (single centre)	41	Mixed	NR	37	NR	0 peri-operative
Veroux 2016 [10]	Italy (single centre)	Cohort	Simultaneous unilateral NN + KT	NR	NR	NR	No graft compromise
Abrol 2021 [45]	USA (Mayo Clinic)	Cohort	Simultaneous bilateral laparoscopic NN + KT	10.5 (median)	NR	NR	Comparable with staged
Grodstein 2017 [31]	USA (single centre, 20 years)	188	Open simultaneous	NR	NR	NR	0 peri-operative
Masterson 2023 [16]	USA (single centre)	14	Robotic	3 (median)	0	0	0
Castaneda 2025 [17]	USA (single centre)	Robotic vs. open	Robotic vs. open	Shorter with robotic	Lower with robotic	Lower with robotic	0

BNN, bilateral native nephrectomy; CD, Clavien–Dindo; Hb, haemoglobin; KT, kidney transplant; LoS, length of stay; MIS, minimally invasive surgery; NN, native nephrectomy; NR, not reported; Tx, transplantation. Direct cross-cohort comparison is limited by differing inclusion criteria, surgical approaches, eras, and complication grading conventions.
